# An Update on Plant Derived Anti-Androgens

**DOI:** 10.5812/ijem.3644

**Published:** 2012-04-20

**Authors:** Paul Grant, Shamin Ramasamy

**Affiliations:** 1 Department of Endocrinology, Kings College Hospital, Denmark Hill, London, UK

**Keywords:** Androgens, Anti-Androgens, Prostate Cancer, Prostatic Hyperplasia, Spearmint

## Abstract

Anti-androgens are an assorted group of drugs and compounds that reduce the levels or activity of androgen hormones within the human body. Disease states in which this is relevant include polycystic ovarian syndrome, hirsutism, acne, benign prostatic hyperplasia, and endocrine related cancers such as carcinoma of the prostate.

We provide an overview and discussion of the use of anti-androgen medications in clinical practice and explore the increasing recognition of the benefits of plant-derived anti-androgens, for example, spearmint tea in the management of PCOS, for which some evidence about efficacy is beginning to emerge. Other agents covered include red reishi, which has been shown to reduce levels 5-alpha reductase, the enzyme that facilitates conversion of testosterone to dihydrotestosterone (DHT); licorice, which has phytoestrogen effects and reduces testosterone levels; Chinese peony, which promotes the aromatization of testosterone into estrogen; green tea, which contains epigallocatechins and also inhibits 5-alpha reductase, thereby reducing the conversion of normal testosterone into the more potent DHT; black cohosh, which has been shown to kill both androgenresponsive and non-responsive human prostate cancer cells; chaste tree, which has a reduces prolactin from the anterior pituitary; and saw palmetto extract, which is used as an anti-androgen although it shown no difference in comparison to placebo in clinical trials.

## 1. Introduction

An androgen antagonist (anti-androgen) can broadly be defined as any compound that has the biological effect of blocking or suppressing the action of male sex hormones such as testosterone within the human body. This may occur at any point in the hypothalamic-pituitary-gonadal-end-organ axis and could be through a direct effect on gonadotropin production at the level of the pituitary or by competing for binding sites at the receptor level on the normally androgen sensitive tissues in the body. Androgens themselves have a diverse range of effects in both males and females and their dysregulation can give rise to a variety of clinical disorders, including polycystic ovarian syndrome, the most common endocrine disorder in females, which affects up to 7% of the population (1); hirsutism; acne vulgaris; prostatic hyperplasia; and male pattern baldness.

There are already several medical treatments that act as androgen antagonists and have recognized uses; however, in recent years, there has been an increasing demand for complementary and alternative therapies, and this has included an interest in the development and use of more plant-derived anti-androgen therapies. This is especially relevant as some medications currently in use have been found to have sub-optimal efficacy in clinical practice, and many patients are keen to try ‘natural’ or ‘alternative’ approaches as opposed to synthetically derived compounds. This review article provides an overview of the conditions, indications, and uses of anti-androgen medications, with a special focus on the renewed interest in the ancient area of plant-derived therapies.

## 2. Methodology

In order to obtain a maximum amount of high quality evidence, we undertook a literature search using the PubMed/Medline and Athens databases using the linked keywords androgens, anti-androgens, phytoestrogens, PCOS, prostate cancer, benign prostatic hyperplasia, spearmint, black cohosh, *Camellia sinensis*, licorice, Chinese peony, chaste tree, and saw palmetto. We were interested in both laboratory and clinical studies as well as in systematic reviews and meta-analyses.

## 3. Indications for the Use of Androgen Antagonists

Anti-androgen medications are of use in a variety of androgen-driven medical and psychological conditions. The most common ones are listed below in Sidebar 1. Anti-androgen therapies in the male can lead to impaired development or reversal of secondary sexual characteristics, reduced libido, testicular atrophy, and erectile and sexual dysfunction. There may also be a corresponding change in other androgen dependent characteristics including muscle bulk and strength, fat mass, male pattern hair growth, skin appearance, energy levels, mood, concentration, and aggression. These are summarized in Sidebar 2 (2).

**Sidebar 1:** Conditions Pertinent for the Use of Anti-Androgens

**Disease state**	Anti-androgen use
**Prostate cancer**	Anti-androgens are useful as anti-neoplastic agents and in palliative, adjuvant or neoadjuvant therapy
**Benign prostatic hypertrophy**	Prostate enlargement
**Male sexual disorders**	Hypersexuality, also known as excessive sexual desire or sexual deviation such as paraphilias
**Acne vulgaris**	To improve skin condition
**Androgenic Alopecia**	Male pattern baldness
**Idiopathic Hirsutism**	Excessive female hairiness
**Polycystic ovarian syndrome**	Regulation of menstrual cycle and reduction of hirsutism
**Gender reassignment therapy**	In male to female transsexuals, anti-androgens are used to suppress the masculinizing effects of androgens
**‘Chemical castration’**	Occasionally anti-androgens are used in registered sex offenders released from prisons to reduce the likelihood of repeat offending by reducing sexual drive

**Sidebar 2:** Androgen Deficiency in the Adult Male

**System**	Signs & Symptoms of androgen deficiency
**Circulation/Central nervous system**	Hot flushes
	Sweats
	Insomnia
	Nervousness
**Mood & cognition**	Irritability & fatigue
	Reduced sense of well being
	Reduced motivation
	Impaired short term memory
	Depression and low self esteem
**Masculinity**	Reduced vigor and physical strength
**Sexuality**	Reduced libido
	Erectile failure
	Impaired orgasm
	Impaired ejaculation and reduced ejaculation volume
**Physical features**	Decreased muscle mass
	Abdominal obesity
	Loss of body hair
**Biochemistry**	Decreased HDL, Increased LDL
	Increased total body fat
	Osteoporosis
	Reduced red cell volume

## 4. An Overview of Current Anti-Androgen Therapies

### 4.1. Cyproterone Acetate

Cyproterone acetate is a synthetically derived steroid that acts as a potent anti-androgen. It also possesses progestational properties and can be used to assist conception in subfertile females.

### 4.2. Spironolactone

Spironolactone, a synthetic 17-spironolactone corticosteroid, is commonly used as a competitive aldosterone antagonist and acts as a potassium sparing diuretic. It used to treat low-renin hypertension, hypokalemia, and Conn’s syndrome. It has recognized anti-androgen effects.

### 4.3. Flutamide/Nilutamide/Bicalutamide

Flutamide/nilutamide/bicalutamide are all non-steroidal, pure anti-androgens. Bicalutamide is the newest agent and has the fewest side effects.

### 4.4. Ketoconazole

Ketoconazole is a derivative of imidazole that is used as a broad spectrum antifungal agent. Recognized effects are severe liver damage, but there is also an adrenolytic function. Ketoconazole reduces androgen production in the testes and the adrenal glands. It is a relatively weak anti-androgen, but is used with good effect in patients with Cushing’s syndrome.

### 4.5. Finasteride/Dutasteride

Finasteride/dutasteride are inhibitors of 5-alpha reductase, an enzyme that prevents the conversion of testosterone into the active form dihydrotestosterone (DHT). They are specific anti-androgens in that they only counteract the effects of testosterone and not other androgens.

### 4.6. Plant-Derived Anti-Androgen Therapies

There is an ever-increasing demand for complementary therapies, or those that are perceived as being more natural. The presence of anti-androgenic chemicals in plants, herbs, and foodstuffs provides an alternative to modern synthetic pharmaceuticals. It is also commonly believed that there are fewer adverse effects of such alternative therapies.

### 4.7. Reishi (Ganoderma lucidum)

Red reishi, commonly known as LingZhi in Chinese, is a mushroom thought to have many health benefits. In a research study exploring the anti-androgenic effects of 20 species of mushrooms, reishi mushrooms had the strongest action in inhibiting testosterone (3). That study found that reishi mushrooms significantly reduced levels of 5-alpha reductase, preventing conversion of testosterone into the more potent DHT. High levels of DHT are a risk factor for conditions such as benign prostatatic hypertrophy (BPH), acne, and baldness.

### 4.8. Licorice (Glycyrrhiza glabra)

Licorice is a flavorful substance that has been used in food and medicinal remedies for thousands of years. It is also known as “sweet root,” licorice root contains a compound that is about 50 times sweeter than sugar. It has been used in both Eastern and Western medicine to treat a variety of illnesses ranging from the common cold to liver disease. Licorice affects the endocrine system because it contains isoflavones (phytoestrogens), which are chemicals found in plants that may mimic the effects of estrogen and relieve menopausal symptoms and menstrual disorders. Licorice may also reduce testosterone levels, which can contribute to hirsutism in women.

A small clinical trial published in 2004 by Armanini and colleagues found that licorice root significantly decreases testosterone levels in healthy female volunteers. Women taking daily licorice root experienced a drop in total testosterone levels after 1 month and testosterone levels returned to normal after discontinuation. It is unclear as to whether licorice root affects free testosterone levels (4). The endocrine effect is thought to be due to phytoestrogens and other chemicals found in licorice root, including the steroid glycyrrhizin and glycyrrhetic acid, which also have a weak anti-androgen effect (5, 6).

### 4.9. White Peony (Paeonia lactiflora)

Chinese peony is a widely grown ornamental plant with several hundred selected cultivars. Many of the cultivars have double flowers with the stamens modified into additional petals. White peony has been important in traditional Chinese medicine and has been shown to affect human androgen levels *in vitro*. In a 1991 study in the American Journal of Chinese Medicine Takeuchi et al described the effects of paeoniflorin, a compound found in white peony that inhibited the production of testosterone and promoted the activity of aromatase, which converts testosterone into estrogen (7). To date, there have been no studies that translate or explore the clinical effects.

### 4.10. Green Tea (Camellia sinensis)

In addition to supporting the cardiovascular system and somewhat reducing the risk of cancer and type 2 diabetes (8), green tea may also have an important anti-androgen effect because it contains epigallocatechins, which inhibit the 5-alpha-reductase conversion of normal testosterone into DHT. As previously noted, this anti-androgen mechanism may help to reduce the risk of BPH, acne, and baldness. As yet, no randomized controlled trials of green tea for these androgen dependent conditions have been conducted.

### 4.11. Spearmint (Mentha spicata [Labiatae])

Spearmint, usually taken in the form of tea, has been thought for many years to have testosterone reducing properties. It is commonly used in Middle Eastern regions as an herbal remedy for hirsutism in females. Its anti-androgenic properties reduce the level of free testosterone in the blood, while leaving total testosterone and DHEAS unaffected, as demonstrated in a study from Turkey by Akdogan and colleagues, in which 21 females with hirsutism (12 with polycystic ovary syndrome and 9 with idiopathic hirsutism) drank a cup of herbal tea steeped with *M. spicata* twice daily for 5 days during the follicular phases of their menstrual cycles. After treatment with the spearmint tea, the patients had significant decreases in free testosterone with increases in luteinizing hormone, follicle-stimulating hormone, and estradiol (9). There were no significant decreases in total testosterone or DHEAS levels. This study was followed by a randomized clinical trial by Grant (10), which showed that drinking spearmint tea twice daily for 30 days (vs. chamomile tea, which was used as a control) significantly reduced plasma levels of gonadotropins and androgens in patients with hirsutism associated with polycystic ovarian syndrome. There was a significant change in patients’ self-reported dermatology-related quality of life indices, but no objective change on the Ferriman-Gallwey scale. It is possible that sustained daily use of spearmint tea could result in further abatement of hirsutism.

### 4.12. Black Cohosh (Actaea racemosa)

Black cohosh (*Actaea racemosa*) is a plant of the buttercup family. Extracts from these plants are thought to possess analgesic, sedative, and anti-inflammatory properties. Black cohosh preparations (tinctures or tablets of dried materials) are used to treat symptoms associated with menopause, such as hot flashes, although the efficacy has been questioned (11). The inhibitory effects of black cohosh extracts (*Cimicifuga* syn. *Actaea racemosa* L.) on the proliferation of human breast cancer cells has been reported recently (12), and Hostsanka. et al (13) have examined the plant’s effects on prostate cancer, another androgen hormone-dependent, epidemiologically important tumor. In that study, the inhibitory effect of an isopropanolic extract of black cohosh (iCR) on cell growth in androgen-sensitive LNCaP and androgen-insensitive PC-3 and DU 145 prostate cancer cells was investigated.

The authors found that regardless of hormone sensitivity, the growth of prostate cancer cells was significantly and dose-dependently down regulated by iCR. At a concentration between 37.1 and 62.7 μg/ml, iCR caused 50% cell growth inhibition in all cell lines after 72h. Increases in the levels of the apoptosis-related M30 antigen of approximately 1.8-, 5.9-, and 5.3-fold over untreated controls were observed in black cohosh-treated PC-3, DU 145, and LNCaP cells, respectively, with the induction of apoptosis being dose- and time-dependent.

Black cohosh extract was therefore shown to kill both androgen-responsive and non-responsive human prostate cancer cells by induction of apoptosis and activation of caspases. This finding suggested that the cells’ hormone responsive status was not a major determinant of the response to the iCR, and indicated that the extract may represent a novel therapeutic approach for the treatment of prostate cancer.

### 4.13. Chaste Tree (Vitex agnus-castus)

Chaste tree (or chasteberry) is a native of the Mediterranean region and is traditionally used to correct hormone imbalances. In ancient times, it was believed to be an anaphrodisiac, hence the name *chaste tree*. Clinical studies have demonstrated effectiveness of medications produced from extract of the plant in the management of premenstrual syndrome (PMS) and cyclical mastalgia (14). The mechanism of action is presumed to be via dopaminergic effects resulting in changes of prolactin secretion from the anterior pituitary. At low doses, it blocks the activation of D2 receptors in the brain by competitive binding, causing a slight increase in prolactin release. In higher concentrations, the binding activity is sufficient to reduce the release of prolactin (15).

Reduction in prolactin levels affects FSH and estrogen levels in females and testosterone levels in men. There is as yet no information regarding its efficacy in endocrine disease states such as PCOS, however, one small-scale study has demonstrated this prolactin reducing effect in a group of healthy males, and the implication is that it could be of use in mild hyperprolactinemia (16, 17). One could also theorize that it could be refined for use as a male contraceptive, because testosterone reduction should reduce libido and sperm production. This topic is further explored in a review by Grant & Anawalt (18).

### 4.14. Saw Palmetto (Serenoa repens)

Saw palmetto is a small palm tree native to eastern regions of the United States. Its extract is believed to be a highly effective anti-androgen as it contains phytoesterols. This has been the subject of a great deal of research with regards to the treatment of BPH (19, 20), androgenic alopecia (21), and PCOS (22). However, controlled trials and other convincing research on its efficacy are still lacking. In the context of BPH, there have been 2 reasonably sized clinical trials that found that saw palmetto extract use showed no difference in comparison to placebo (23, 24). In meta-analyses, it has been shown to be safe and effective in mild to moderate BPH when compared to finasteride, tamsulosin, and placebo (25, 26). However, a more recent meta-analysis showed that it is only superior specifically with regards to the symptom of nocturia (27). Therefore, evidence for its routine use is far from convincing and additional research is necessary to determine its true effectiveness.

## 5. Discussion

There is clearly a need for a greater variety and more efficacious drugs to treat androgen related disorders such as those outlined above. The fact that there is an increasing emphasis over recent years on the potential for alternative anti-androgen compounds derived from plants is interesting and reflects the fact that from a clinical perspective the medications that are used in practice tend not to work very well for the majority of patients, and it can takes several medication changes and dose adjustments to find a routine that works for an individual patient. The research that has been undertaken, which is summarized in Sidebar 3, offers hope that alternative treatment options are available and may produce clinically effective therapies in the future, once suitably refined. Multiple challenges remain, however. Firstly the number of experimental and clinical studies remains relatively small and secondly they are often limited in terms of their quality (lack of adequate numbers to achieve statistical significance, lack of randomized controlled trials, and the findings that these compounds only appear to have marginal efficacy when assessed in head-to-head clinical trials). One extremely good review from the Cochrane group examined the use of *Serenoa repens* in 9 clinical trials. This is a popular herbal medicine for BPH and the review found that it was well tolerated, but was no better than placebo in improving urinary symptom scores. Nor did *Serenoa repens* provide noticeable relief, generally considered to be a decrease of 3 points, in urinary symptoms (27). To date, there have been relatively few high quality long-term randomized studies evaluating standardized preparations of (potentially) clinically relevant doses. Given the frequent use of *Serenoa repens* and the relatively low quality of existing evidence, a few more well designed, randomized, placebo-controlled studies that are adequately powered, use validated symptom-scale scores, and have a placebo arm and a minimum follow-up of 1 year, are needed to confirm, or deny, these findings. The same argument can be made for all of the plant-derived anti-androgens covered in this review. While there may be detectable and significant biochemical and *in vitro* changes in androgen related parameters, we are still not clear as to the benefits in endocrine practice.

**Sidebar 3:** Summary of Effects of Plant Derived Anti-Androgens

Plant Derived Anti-Androgen	Clinical/Biological Effects	Reference
**Red Reishi (*Ganoderma lucidum*)**	Reduction in 5-alpha-reducatase enzyme activity, reduction in DHT levels	(3)
**Licorice (*Glycyrrhiza glabra*)**	Reduction in total testosterone levels (effect on free testosterone levels not clear)	(4-6)
**White Peony (*Paeonia lactiflora*)**	Paeoniflorin inhibits the production of testosterone and promotes the activity of aromatase - the enzyme that converts testosterone into estrogen	(7)
**Green Tea (*Camellia Sinensis)***	Contains chemicals epigallocatechins, which inhibit the enzyme 5-alpha-reductase, and thereby reduce the conversion of normal testosterone into the more potent DHT	(8)
**Spearmint (*Mentha spicata*[Labiatae])**	Decreases free testosterone, increases LH, FSH and estradiol. Reduction in patient reported measures of hirsutism	(9, 10)
**Black Cohosh (*Actaea racemosa)***	Black cohosh extract has been shown to inhibit the proliferation of human breast cancer cells and kill both androgen-responsive and unresponsive human prostate cancer cells by induction of apoptosis and activation of caspases.	(11-13)
**Chaste Tree (*Vitex agnus-castus*)**	Clinical studies have demonstrated effectiveness of medications produced from extract of the plant in the management of premenstrual syndrome (PMS) and cyclical breast pain (mastalgia) as well reduction of mild hyperprolactinemia.	(14-17)
**Saw Palmetto (*Serenoa repens)***	Shown to be of efficacy for the treatment of nocturia in the context of BPH only	(19, 20, 25-27)

## 6. Conclusions

The essence of endocrinology is to suppress overactivity and stimulate or replace hypofunction in order to restore normality to the body’s hormonal axes. Androgen dysregulation is a feature of several common, and not so common, disease processes. In terms of male sexual dysfunction, the etiology, pathophysiology, and implications are often complex (28). Targeting drug compounds to block the actions and effects of androgens can be challenging and the standard treatments often have variable rates of success.

Alternative medicine is any healing practice that does not fall within the realm of “conventional” medicine. Reports of its efficacy are often anecdotal and based on historical or cultural traditions, rather than on scientific evidence. For the most part, the plant derived anti-androgen therapies discussed above used to fall into this category. However, there is now a small amount of data showing that androgen, prolactin, and gonadotropin levels can be biochemically modulated by the active compounds contained within these natural sources. Further work is clearly needed before the use of such compounds becomes part of routine practice. There is currently a paucity of high quality data derived from rigorously conducted trials. However, there are a few clinical and pre-clinical studies, although small in number, that have demonstrated that some natural anti-androgens address the underlying pathophysiology and can have effects on endocrine mediated disorders. What is now required are several larger, well-controlled, randomized studies aimed at proving their efficacy. While this group of treatments may be slow to find favor and may not be used first line, it does at least appear to be more acceptable to patients because of its perceived more natural origins (29).

## References

[1] Grant P Polycystic ovary syndrome.. http://www.yourhormones.info/endocrine_conditions/polycystic_ovary_syndrome.aspx.

[2] Lee OD (2011). Think androgen deficiency.. Am J Mens Health..

[3] Fujita R, Liu J, Shimizu K, Konishi F, Noda K, Kumamoto S (2005). Anti-androgenic activities of Ganoderma lucidum.. J Ethnopharmacol..

[4] Armanini D, Bonanni G, Palermo M (1999). Reduction of serum testosterone in men by licorice.. N Engl J Med..

[5] Somjen D, Knoll E, Vaya J, Stern N, Tamir S (2004). Estrogen-like activity of licorice root constituents: glabridin and glabrene, in vascular tissues in vitro and in vivo.. J Steroid Biochem Mol Biol..

[6] Tamir S, Eizenberg M, Somjen D, Izrael S, Vaya J (2001). Estrogen-like activity of glabrene and other constituents isolated from licorice root.. J Steroid Biochem Mol Biol..

[7] Takeuchi T, Nishii O, Okamura T, Yaginuma T (1991). Effect of paeoniflorin, glycyrrhizin and glycyrrhetic acid on ovarian androgen production.. Am J Chin Med..

[8] Grant P, Dworakowska D, Preedy V (2012). Tea and Diabetes: the laboratory and the real world.. Tea in Health & Disease Prevention..

[9] Akdogan M, Tamer MN, Cure E, Cure MC, Koroglu BK, Delibas N (2007). Effect of spearmint (Mentha spicata Labiatae) teas on androgen levels in women with hirsutism.. Phytother Res..

[10] Grant P (2010). Spearmint herbal tea has significant anti-androgen effects in polycystic ovarian syndrome. A randomized controlled trial.. Phytother Res..

[11] Newton KM, Reed SD, LaCroix AZ, Grothaus LC, Ehrlich K, Guiltinan J (2006). Treatment of vasomotor symptoms of menopause with black cohosh, multibotanicals, soy, hormone therapy, or placebo: a randomized trial.. Ann Intern Med..

[12] Fang ZZ, Nian Y, Li W, Wu JJ, Ge GB, Dong PP (2011). Cycloartane triterpenoids from Cimicifuga yunnanensis induce apoptosis of breast cancer cells (MCF7) via p53-dependent mitochondrial signaling pathway.. Phytother Res..

[13] Hostanska K, Nisslein T, Freudenstein J, Reichling J, Saller R (2005). Apoptosis of human prostate androgen-dependent and -independent carcinoma cells induced by an isopropanolic extract of black cohosh involves degradation of cytokeratin (CK) 18.. Anticancer Res..

[14] Daniele C, Thompson Coon J, Pittler MH, Ernst E (2005). Vitex agnus castus: a systematic review of adverse events.. Drug Saf..

[15] Webster DE, He Y, Chen SN, Pauli GF, Farnsworth NR, Wang ZJ (2011). Opioidergic mechanisms underlying the actions of Vitex agnus-castus L.. Biochem Pharmacol..

[16] Azadbakht M, Baheddini A, Shorideh S, Naser Zadeh A (2005). Effect of vitex agnus-castus l. leaf and fruit flavonoidal extracts on serum prolactin concentration.. J Med Plants..

[17] Merz PG, Gorkow C, Schrodter A, Rietbrock S, Sieder C, Loew D (1996). The effects of a special Agnus castus extract (BP1095E1) on prolactin secretion in healthy male subjects.. Exp Clin Endocrinol Diabetes..

[18] Grant NN, Anawalt BD (2002). Male hormonal contraception: an update on research progress.. Treat Endocrinol..

[19] Boyle P, Robertson C, Lowe F, Roehrborn C (2004). Updated meta-analysis of clinical trials of Serenoa repens extract in the treatment of symptomatic benign prostatic hyperplasia.. BJU Int..

[20] Wilt T, Ishani A, Mac Donald R (2002). Serenoa repens for benign prostatic hyperplasia.. Cochrane Database Syst Rev..

[21] Murugusundram S (2009). Serenoa Repens: Does It have Any Role in the Management of Androgenetic Alopecia?. J Cutan Aesthet Surg..

[22] Liepa GU, Sengupta A, Karsies D (2008). Polycystic ovary syndrome (PCOS) and other androgen excess-related conditions: can changes in dietary intake make a difference?. Nutr Clin Pract..

[23] Bent S, Kane C, Shinohara K, Neuhaus J, Hudes ES, Goldberg H (2006). Saw palmetto for benign prostatic hyperplasia.. N Engl J Med..

[24] Dedhia RC, McVary KT (2008). Phytotherapy for lower urinary tract symptoms secondary to benign prostatic hyperplasia.. J Urol..

[25] Geavlete P, Multescu R, Geavlete B (2011). Serenoa repens extract in the treatment of benign prostatic hyperplasia.. Ther Adv Urol..

[26] Sosnowska J, Balslev H (2009). American palm ethnomedicine: a meta-analysis.. J Ethnobiol Ethnomed..

[27] Tacklind J, MacDonald R, Rutks I, Wilt TJ (2009). Serenoa repens for benign prostatic hyperplasia.. Cochrane Database Syst Rev..

[28] Grant P, Ramasamy S, Grant P (2012). The Pituitary Gland & Erectile Dysfunction. Erectile Dysfunction: Causes, Risk Factors & Management..

[29] Magin PJ, Adams J, Heading GS, Pond DC, Smith W (2006). Complementary and alternative medicine therapies in acne, psoriasis, and atopic eczema: results of a qualitative study of patients' experiences and perceptions.. J Altern Complement Med..

